# A curious case of de novo anti-HLA-C antibody-mediated humoral rejection and Fabry-like zebra bodies in a renal transplant recipient 

**DOI:** 10.5414/CNCS109998

**Published:** 2020-01-30

**Authors:** Mohammad Abuzeineh, Ahmad Ziadeh, Taba Kheradmand, Van Nguyen, Preethi Yerram

**Affiliations:** 1 Division of Nephrology, University of Missouri, Columbia, MO,; 2 Midwest Transplant Network, Westwood, KS, and; 3 Department of Pathology and Anatomical Sciences, University of Missouri, Columbia, MO, USA

**Keywords:** HLA-C antibody-mediated renal transplant rejection, zebra bodies in renal transplant

## Abstract

Detection of donor-specific antibodies (DSA) is an essential part of diagnosing antibody-mediated renal allograft rejection (ABMR). The role of solitary preformed, or post-transplant HLA-C antigens in solid organ transplantation is unclear, due to the less sensitive nature of the historical assays, lack of data, low expression level on the cell surface, and their co-existence with other anti-HLA DSA. Herein, we present the case of a 39-year-old African American man, without prior history of pre-transplant sensitization that was diagnosed with biopsy-proven ABMR due to de novo donor-specific anti-HLA-C antibodies. This case report illustrates the role of HLA-C antibodies in causing ABMR if generated toward immunogenic-shared epitopes and demonstrates the need for their recognition in the pre- and post-transplant period. Another interesting aspect of this case is the incidental finding of Fabry-like zebra bodies, which we eventually determined to be of unclear etiology.

## Introduction 

Antibody-mediated renal allograft rejection (ABMR) is a well-known cause of allograft loss in renal transplant recipients. Its diagnosis requires histological evidence of acute tissue injury, circulating donor-specific antibodies (DSA), and immunologic evidence of an antibody-mediated process (such as C4d deposition in the allograft). DSA are detected in most cases of ABMR in which diffuse peritubular capillaries (PTC) C4d is positive [1, 2]. Although HLA-C matching is not taken into consideration when assessing histocompatibility and matching in kidney transplantation due to the close linkage disequilibrium with HLA-B antigens as well as its low expression level, recent reports have shown that antibodies targeted towards pre-existing immunogenic epitope of HLA-C antibodies are associated with allograft rejection and graft failure [3, 4, 5, 6]. In this context, we present a patient with biopsy features of ABMR, who on further testing showed strong DSA to an immunogenic epitope of HLA-C7. In addition, his renal biopsy showed Fabry-like ultrastructural “zebra bodies” on electron microscopy (EM). 

## Case presentation 

A 39-year-old African American male with end-stage renal disease (ESRD) presumed to be secondary to hypertensive nephropathy underwent 2A/2B/2DR mismatched, CMV +/+, deceased donor kidney transplantation in 2014. Flow cytometry crossmatch was negative. He received induction immunosuppression with anti-thymocyte globulin (rabbit) 6 mg/kg and intravenous (IV) steroid and started on triple immunosuppressive drug regimen with tacrolimus, mycophenolic acid (MPA), and prednisone. He had an uncomplicated post-transplant course, and his creatinine (Cr) stabilized around 1.4 – 1.6 mg/dL. 

**Figure 1.**
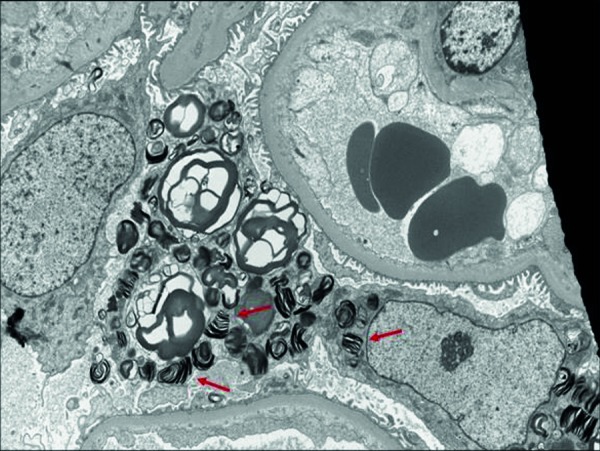
EM red arrows: Incidental zebra pattern lipid inclusions present in podocytes.

The patient’s home immunosuppression regimen was tacrolimus 2 mg twice a day (b.i.d.), MPA 360 mg b.i.d., and prednisone 5 mg once daily. During a routine follow-up visit 43 months post transplant, while asymptomatic, the patient’s serum Cr was noted to be acutely elevated at 2.16 mg/dL compared to his baseline of 1.4 – 1.6 mg/dL. His serum tacrolimus level was 5.7 ng/mL (within our target therapeutic range 4 – 6 ng/mL), and the patient did not have any hematuria or proteinuria on urinalysis. A renal allograft biopsy was obtained which showed glomerular changes suspicious for early transplant glomerulopathy (Banff score cg1b) with scattered thickening and duplication of glomerular basement membrane (GBM) without diagnostic evidence of acute cellular or humoral rejection. There were scattered peritubular capillaries with increased intravascular lymphocytic infiltrate (Banff score ptc1) with no definitive evidence of endotheliitis of the arteries (Banff score v0). The C4d staining on immunofluorescence (IF) was weak and focal in PTC and GBM (Banff score C4d 1). The biopsy also showed incidental finding of ultrastructural zebra-patterned lipid inclusions in podocytes on EM, initially raising suspicion for donor-derived Fabry’s disease (Figure 1). Based on these biopsy findings, changes were thought to be chronic, and we planned to continue with his current immunosuppression regimen and ensure compliance. 

Five days after the biopsy, the patient presented to the emergency department (ED) with headache, dyspnea, and oliguria, with blood pressures as high as 214/97. His serum Cr was found to be further elevated to 3.10 mg/dL, urine testing showed microscopic hematuria and nephrotic range proteinuria of 4 g/day. The patient was given a dose of IV furosemide 80 mg in the ED for concern of pulmonary edema, and admitted to hospital. The initial impression was possible hypertensive emergency leading to acute kidney injury (AKI) of the renal allograft. CMV and BK virus PCR were negative along with normal complement levels. His blood pressure was controlled over a few days, however his renal function continued to worsen in the setting of oliguria. In the context of these developments, the patient was suspected to have acute rejection. Serum was tested for presence of DSA, followed by a repeat renal allograft biopsy (second biopsy). The patient was empirically started on IV steroid pulse therapy, and his MPA dose was increased to 720 mg b.i.d., without improvement in renal function. Repeat renal allograft biopsy showed capillaritis (Banff score ptc2) and arteritis with focal fibrinoid necrosis (Banff score v3), glomerulitis (Banff score g3) with moderately increased cellularity within the capillary loops with predominantly mononuclear cells, endothelial cells were swollen, and weak and focal C4d deposits on IF involving more than 50% of PTC (Banff score C4d2-3) concerning for active ABMR, without evidence of tubulitis or significant interstitial inflammation. Biopsy also showed similar transplant glomerulopathy (Banff score cg1b) mentioned in first biopsy, and interstitial fibrosis was estimated ~ 10%. No diagnostic evidence of microthrombi was seen within the capillary loops making thrombotic microangiopathy unlikely. 

**Figure 2.**
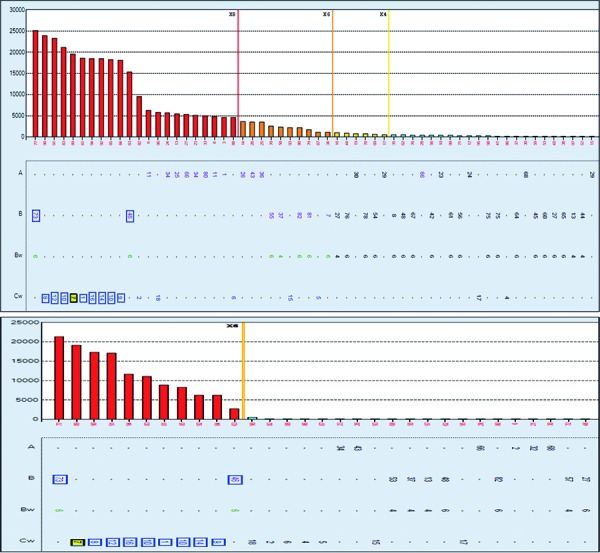
Top panel shows anti-HLA-C antibodies to C1, 7, 8, 9, 10, 12, 14, and 16 as well as B46 and 73 with the same pattern of antibody reactivity present on the C1q binding assay (bottom panel). IgG and C1q-fixing HLA antibodies were detected using the single antigen luminex solid phase assay (One Lambda, Thermo Fisher, CA, USA). HLAMatchmaker DRDQDP (version 2.2) was used to identify antibody specificities to a shared epitope, 76VRN, of the detected antibodies.

Post-transplant antibody screening showed strong DSA to C7 (mean fluorescent intensity (MFI) of 19,600) and moderate DSA to C2 (MFI of 9,550).This was the first post-transplant sample tested for presence of DSA, and therefore no previous antibody testing was available to assess the progression of de novo DSA development since transplant. Interestingly the serum antibody screening revealed a strong pattern of anti-HLA-C antibodies to C1, 7, 8, 9, 10, 12, 14, and 16 as well as B46 and 73 (Figure 2, top panel). In addition, the same pattern of antibody reactivity was present on the C1q binding assay (Figure 2, bottom panel) consistent with C4d-positive histopathology findings. HLA epitope analysis revealed reactivity to the antibody-verified 76VRN epitope [14] that is shared among these HLA antigens and is different from self-epitope 76VRK at position 78 with a lysine instead of an Asparagine. Interestingly, EM of the second biopsy did not show the ultrastructural zebra bodies. 

In view of these findings, the patient was treated as a case of active ABMR and was started on a course of therapeutic plasma exchange (TPE) and intravenous immunoglobulin (IVIG) 100 mg/kg post each TPE session. However, after the second TPE session, the patient had acute hypoxic respiratory failure requiring intubation and mechanical ventilation thought to be secondary to hospital-acquired pneumonia and pulmonary edema. The patient was started on hemodialysis (HD) due to oliguric AKI. 

The patient went on to complete five sessions of TPE, with IVIG administration after each session. He eventually improved clinically, but remained dialysis-dependent. Risks and benefits of further anti-rejection therapy were considered, and a decision was made to repeat renal allograft biopsy (third biopsy) to evaluate the degree of interstitial fibrosis, and to check if the renal allograft is salvageable. 

The third renal allograft biopsy showed more diffuse transplant glomerulopathy (Banff score cg3), with much improved peritubular capillaritis (Banff score ptc0-1) and ~ 10% interstitial fibrosis. The amount of inflammation of peritubular capillaries was noted to be significantly better (80% improvement) as compared to the previous biopsy (Banff score ptc0-1, v1). However, the arterioles showed significant intimal fibrosis compared to the previous biopsy, and C4d deposits on IF were still noted to be weak (Banff score C4d1). Table 1 lists the Banff score summary of the three biopsies. Repeat HLA antibody screening showed persistence of DSA to C7 (MFI = 11,500) with the same pattern of reactivity to the 76VRN immunogenic epitope, although at relatively weaker levels. In view of these findings, the patient was given IV rituximab at a dose of 375mg/m^2^, and his prednisone dose was increased to 60 mg, with plan for gradual tapering. He was maintained on tacrolimus 4 mg b.i.d. (based on serial trough levels), while MPA was continued at 360 mg b.i.d.. The patient was subsequently discharged home, but he remained dialysis-dependent. 

## Discussion 

ABMR is frequently attributed to DSA against HLA-A, B, DR, and DQ antibodies. Rejection due to the presence of DSA to isolated HLA-C antigens is uncommon, and the lower levels of cell surface expression of HLA-C antigens in comparison to HLA-A, B, and DR may have contributed to the lack of historical data on significance of HLA-C in ABMR [7]. HLA-C-presented antigens are recognized by cytotoxic T lymphocytes [8, 9] and can induce an antibody response [10], although at lower levels, in comparison to the HLA-A and B loci [11, 12, 13]. Nonetheless, the clinical relevance of these antibodies has not been well established. More recently, several case reports have implicated HLA-C antibodies in causing ABMR. Suneja et al. [3] described ABMR associated with HLA-C17 DSA, 21 months after transplantation. Similar findings were described by Bachelet et al. [4] and Bosch et al. [5]. Although most reports lack analysis of antibody generation to immunogenic public epitopes of HLA-C antigens, Persaud et al. [6] presented the case of an accelerated acute humoral response due to a C14 antigen bearing a Bw6 epitope at position 80NRG, which the patient was previously sensitized to through pregnancy. In our patient’s case, the antibody to HLA-C7 generated to the 76VRN public epitope, is shared by several HLA-C as well as HLA-B antigens; however, this epitope is different from the Bw6 public epitope-associated antibody described by Persaud et al. [6]. As in our case, the Bw6 is considered a self-epitope due to the patient’s B35 antigen. These findings demonstrate the importance of HLA-C antigen in humoral allosensitization and ABMR, and emphasize the importance of epitope analysis in understanding the antibody response generated towards the intra- or inter-locus immunogenic public epitopes [13]. 

The other interesting aspect of this case report is the presence of focal ultrastructural zebra-patterned lipid inclusions in podocytes in the first renal biopsy, which raised suspicion for donor-derived Fabry’s disease. Fabry’s disease is an X-linked inborn error of the glycosphingolipid metabolic pathway that results in lysosomal accumulation of globotriaosylceramide (Gb3) in a wide variety of cells, leading to the various manifestations of the disease [15]. Its renal involvement is confirmed by EM which classically shows deposits of Gb3 that appear primarily within enlarged secondary lysosomes as lamellated membrane structures, called myeloid or zebra bodies [16]. The focal presence of these myeloid bodies on the first biopsy and their absence on the subsequent biopsy in our patient makes Fabry’s disease unlikely, as these findings are usually diffuse and consistent. However, the donor was a female who could have been heterozygous for Fabry’s disease, and interestingly, Mauer et al. [17] described mosaicism in podocyte involvement in females with Fabry’s disease, which could explain the discrepancy in biopsies. The patient and his family members tested normal for enzyme activity (α-galactosidase A enzyme). Other known causes for similar findings include hydroxychloroquine, which, by causing phospholipidosis, can mimic zebra bodies, and be mistaken for Fabry’s disease. This was evident in multiple case reports [18, 19, 20]. 

Our patient was not taking hydroxychloroquine; and for the donor’s privacy we could not pursue further information about the donor nor her family. Thus we determined the first biopsy findings to be of unclear etiology (artefactual, drug induced – other than hydroxychloroquine –, female donor with undiagnosed heterozygous Fabry’s disease, or others). 

## Funding 

There are no funding statements for this case study. 

## Conflict of interest 

All authors have no conflict of interest to declare. 

**Table 1.** Banff scores of the three transplant kidney biopsies. The first biopsy was performed on day 1.

Biopsy	Day	ptc	g	c4d	v	t	i	cg	Grade
First	1	1	1 – 2	1	0	1	0	1b	1
Second	13	2	3	2 – 3	3	1	0	1b	1
Third	37	0 – 1	2	1	1	1	0	3	1
